# Surface CD69‐Negative CD4 and CD8 Bone Marrow‐Resident Human Memory T Cells

**DOI:** 10.1002/eji.202451529

**Published:** 2025-05-16

**Authors:** Emilia Schneider Revueltas, Marta Ferreira‐Gomes, Gabriela Maria Guerra, Pawel Durek, Frederik Heinrich, Anna Casanovas Subirana, Koji Tokoyoda, Jun Dong, Simon Reinke, Sebastian Hardt, Christian Hipfl, Thomas Dörner, Carsten Perka, Ute Hoffmann, Hyun‐Dong Chang, Mir‐Farzin Mashreghi, Andreas Radbruch

**Affiliations:** ^1^ Deutsches Rheuma‐Forschungszentrum Berlin ein Institut der Leibniz Gemeinschaft Berlin Germany; ^2^ Tottori University Yonago Japan; ^3^ Berlin Institute of Health Charité‐Universitätsmedizin Berlin and BIH Center for Regenerative Therapies (BCRT) Berlin Germany; ^4^ Department of Orthopedic Surgery Charité‐Universitätsmedizin Berlin Berlin Germany; ^5^ Department of Rheumatology and Clinical Immunology Charité‐Universitätsmedizin Berlin Berlin Germany; ^6^ Technische Universität Berlin Berlin Germany

**Keywords:** bone marrow, CD69, memory T lymphocytes, T cell receptor repertoire, transcriptome

## Abstract

Across tissues, tissue‐resident memory T cells have been defined as cells that express CD69 on their cell surface but not sphingosine‐1‐phosphate receptor 1 (S1PR1), the receptor for the tissue‐egress signal sphingosine‐1‐phosphate (S1P). It is less clear whether CD69‐negative memory T cells are also tissue‐resident. Here, we compare transcriptomes and T cell receptor repertoires of individual CD4 and CD8 memory T cells from paired blood and bone marrow samples from three human donors. CD69^−^ memory T cells of blood and bone marrow share transcriptionally defined clusters, characterized by signature genes and reflecting their imprinting during original activation. However, cells of related clusters from blood and bone marrow have different TCR repertoires, evidence that they represent distinct compartments of memory and indicating that the CD69^−^ memory T cells are residents of the bone marrow. Interestingly, the surface CD69^−^ memory T cells of bone marrow do transcribe the CD69 gene and express S1PR1, suggesting that they are blindfolded to the perception of the egress signal sphingosine‐1‐phosphate by dimerization and internalization of CD69 and S1PR1, maintaining them in the bone marrow.

## Graphical abstract

Through the analysis of TCR repertoires, differential gene expression, and flow cytometry, we propose a mechanism in which surface CD69‐negative memory T cells reside in the bone marrow of healthy humans. Therefore, we challenge the notion of surface CD69‐positive T cells representing the only bona fide resident phenotype in the bone marrow.

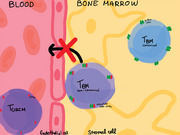

**Abbreviations**
T_BL_
: memory T cells of the blood
T_BM_
: memory T cells of the bone marrow
TCR: T‐cell receptor
T_M_
: memory T cells

## 1 Introduction

Tissue‐resident memory T cells are considered key to local and systemic immunity [1, 2, 3, 4, 5], although their relation to memory T cells circulating in blood and lymph is less well‐defined. It is mostly accepted that memory T cells expressing CD69 on their cell surface, but not the receptor for sphingosine‐1‐phosphate, S1PR1, a signal for egress into the blood, are truly resident memory T cells [3, 5, 6, 7, 8, 9]. It has remained a question of continued discussion whether memory T cells found in tissues that do not express CD69 on their cell surface are residents of the tissue or rather cells circulating through that tissue [10].

About 30% of all CD45RO^+^ CD4^+^ memory T cells and 60% of all CD45RO^+^ CD8^+^ memory T cells in the human bone marrow express CD69 on the cell surface. It should be noted that while CD69^+^ memory T cells of the bone marrow do not transcribe the S1PR1 gene, in contrast, CD69^−^ cells transcribe both the S1PR1 gene [3] and the transcription factor Krüppel‐like factor 2 (KLF2), inducing its expression. On the other hand, all CD4^+^ and CD8^+^ memory T cells of the bone marrow uniformly and individually co‐localize to stromal cells expressing interleukin 7 (IL‐7), as shown for the mouse [1, 11–13] suggesting that CD69^+^ and CD69^–^ memory T cells of the bone marrow have a similar lifestyle and might both be residents of the bone marrow.

Here, we present single‐cell transcriptomes and T cell receptor (TCR) repertoires of CD4^+^ and CD8^+^ memory T cells from paired blood (T_BL_) and bone marrow (T_BM_) samples from three human donors. Among the cells of different origins, surface CD69^+^ and CD69^−^ expressing cells were separated using simultaneous cellular indexing of transcriptomes and epitopes by sequencing (CITE‐Seq). Transcriptome signatures identified about 10 clusters of CD4 and CD8 memory T cells each, mostly according to the expression of genes related to their imprinting when originally activated. When comparing TCR repertoires of surface CD69^+^ and surface CD69^−^ T_BM_ and CD69^−^ T_BL_ of transcriptionally similar cells, we find significantly different repertoires for most cases. These exclusive repertoires define them as belonging to separate compartments and indicate the residency of CD69^−^ bone marrow memory T cells. Moreover, we show that surface CD69‐negative bone marrow memory cells actually transcribe higher amounts of the CD69 gene, in contrast to their counterparts from the blood. Since it is known that CD69 interacts with S1PR1, causing internalization of the receptor [14, 15], this observation provides a potential molecular mechanism explaining the residency of CD69^−^ memory T cells in the bone marrow.

## 2 Results

### 2.1 Transcriptional Landscape of Memory T Cells from Blood and Bone Marrow

**FIGURE 1:**
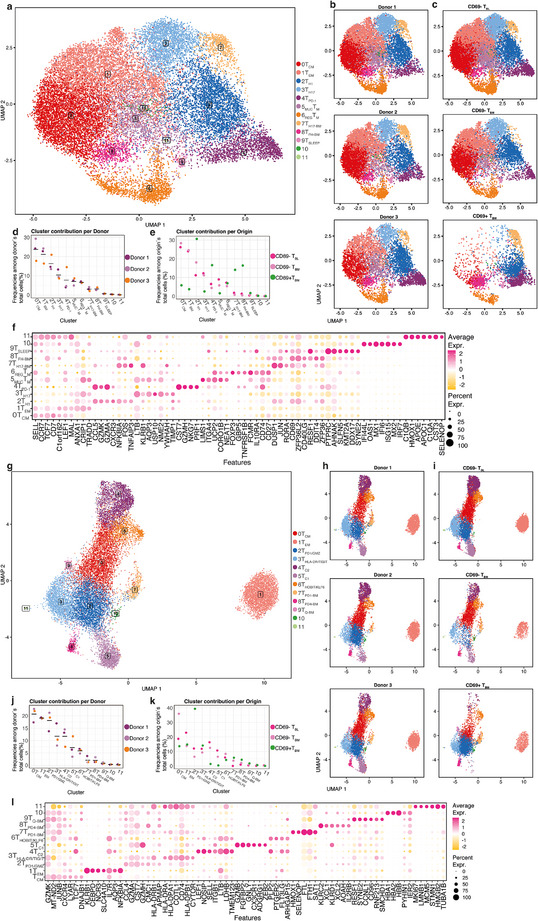
Transcriptional landscape of TCR+ CD4+ and CD8+ memory T cells. Cells from paired blood and bone marrow of three donors were isolated by MACS and sorted by FACS for single‐cell RNA sequencing (gating strategy and experimental workflow scheme in Figure S1A,B). To note, (A)–(F) belong to 19279 CD4^+^ TCR^+^ memory T cells, while (G)–(L) to 18651 CD8^+^ TCR^+^ memory T cells. (A, G) UMAP representation of all cells together, (B, H) shows UMAPs of cells from individual donors and (C, I) of different tissues and CD69 cell surface expression, as defined by CITE‐seq. Each dot represents a cell, and the color code corresponds to the cluster to which they were assigned. Clusters of transcriptionally similar cells were defined by shared nearest neighbor (SNN) modularity optimization. (D, J) Frequencies of clustered memory T cells normalized to the donor's total cell numbers. The horizontal line indicates the median value. (E, K) Frequencies of clustered memory T cells normalized to tissue and CD69 cell surface expression. (F, L) Gene expression signatures of the top 7 significantly differentially expressed genes. Color indicates the average expression of a gene in comparison to the other clusters, and the bubble size indicates the frequencies of cells expressing the gene. At least 50% of the cells of a cluster express any of the marker genes, with more than 0.25 log‐fold‐change (Seurat).

Here, we describe the T cell antigen‐receptor repertoire and transcriptomes of 19279 CD4^+^ and 18651 CD8^+^ CD45RO+ memory T cells (Tm) from paired human blood and bone marrow samples collected from three individuals undergoing hip joint‐replacement surgery (Table S1). The cells were isolated as described in Figure S1A and incubated in addition with DNA‐barcoded antibodies for Cellular Indexing of Transcriptomes and Epitopes (CITE‐Seq). Sorted CD4^+^ and CD8^+^ Tm cells were then subjected to single cell transcriptome, as well as full‐length T‐cell antigen receptor (TCR) sequencing. Cells were grouped according to their expression of CD4 and CD8A transcripts into CD4^+^ and CD8^+^ subsets, respectively. Only cells for which the TCR sequence was obtained were considered for further analysis. Cells were clustered according to their transcriptome similarity, and clusters were visualized by uniform manifold approximation and dimension reduction (UMAP) [16]. For CD4^+^ and CD8^+^ memory T cells, 12 subpopulations each were obtained (Figure 1A,G) for all three donors (Figure 1B,G). Cells were classified as CD69+ or CD69^−^ according to the labelling of surface CD69 by CITE‐Seq (Figure S1C). The distribution of cells in the clusters was similar between CD69^−^ Tm from blood and CD69^−^ Tm from bone marrow (T_BM_) but different for CD69^+^ T_BM_ (Figure 1C,I).

The identity of each cluster, as revealed by the characteristic gene expression signature, is shown in Figure 1F,L for CD4^+^ and CD8+ T_M_, respectively. Selected genes that are significantly differentially expressed are shown in Tables 1 and 2. The frequencies of cells in each cluster are shown in Figure 1D,J and Tables 1 and 2. Based on their gene expression signature, we identified CD4^+^ T_M_ cells in **cluster 0** to be central memory (T_CM_) cells [5, 17]. Frequencies differed substantially between CD69^−^ T_BL_ and T_BM_, and CD69^+^ T_BM_ (Table 1). CD4^+^ memory T cells found in **cluster 1** (Table 1) expressed SELL, TCF7, and LEF1, like those of cluster 0. They did not express CCR7 and CD7, but did express CRIP, ANXA1, S100A11 and VIM, a signature classifying them as effector memory cells (T_EM_) [18]. Memory T cells in **cluster 2** expressed CCL5 (RANTES) and CXCR3, hallmarks of instruction by interferon‐γ [19] and T_H1_ memory cells [20]. They also expressed GZMK and GZMA, a signature of T_H1_ memory cells with cytotoxic potential [21]. These T_H1_ cells were the predominant cluster of CD69^+^ T_BM_. Interestingly, CD69^−^ T_BM_ in cluster 2 transcribed the CD69 gene, although they did not express it on the cell surface (Figure S1C; Figure 3C; Figure S3A,B). Cells in **cluster 3** expressed RORC, KLRB1, and CCR6, classifying them as T_H17_ memory cells [22]. Cells in **cluster 4**, which we annotated as T_PD‐1_, expressed the gene signature of resting Tr1 cells with regulatory potential, expressing granzyme genes and PD‐1 [21, 23] (Table 1; Figure S5). These cells have been described in patients with juvenile idiopathic arthritis [24]. They are also described in the accompanying paper of Pulvirenti as EOMES+Tr1‐like cells, which we also find in cluster 2 [25]. Cells in **cluster 5** expressed ITGA4 and ITGB7, that is, the integrin LPAM1, binding to MAdCAM‐1, and pointing to mucosal affiliation [26]. These “mucosal Tm” (_MUC_T_M_) made up 8.9% of CD4^+^ T_BL_ and 5.8% of CD69^−^ T_BM_ but were absent from CD69^+^ T_BM_ (1.5%). Cells in **cluster 6** expressed FOXP3, classifying them as regulatory Tm (_REG_T_M_). Cells in **clusters 7** and **8** were essentially absent from blood (1.8 and 1.3%) and CD69^−^ T_BM_ (0.8 and 0.5%). We named the **cluster 7** memory cells T_H17‐BM_ since they did express RORC. Cells of **cluster 8** (T_FH‐BM_) expressed CD40LG and thus qualified as follicular helper memory cells. They also expressed ZFP36 family transcription factors described to be involved in maintaining T cell quiescence [27, 28]. They expressed JUN and FOS of the AP‐1 transcription factor family, involved in the maintenance of tissue‐resident T_M_ [29], and RORA, involved in the maintenance of ILC1 memory cells [30]. Cells in **cluster 9** made up less than 1% among CD4^+^ T_BL_ and CD69^−^ T_BM_ and 1.7% among CD69^+^ T_BM_. We named these cells “sleepy” T_BM_ since they expressed BTG1, BTG2, inhibiting proliferation [31], and SLFN5, with “Schlafen” in its name [32]. Cells in **cluster 10** represented 0.3% of CD4+ T_BL_, 0.3% of CD69^−^ T_BM_, and 0.2% of CD69^+^ T_BM_. We did not consider cells of clusters 10 or **cluster 11** for further analysis due to the low absolute cell numbers in these clusters.

**TABLE 1:** TCR^+^ CD4^+^ memory T cell gene expression signatures and frequencies.

CD4 Cluster	Annotation	Blood CD69^−^	BM CD69^−^	BM CD69^+^	Signature genes
0	T_CM_	26.2	28.3	5.8	SELL, CCR7, TCF7, CD7, LEF1
1	T_EM_	24.0	24.6	3.7	SELL, TCF7, ANXA1, TAGLN2, CRIP1, MAL, TRADD, S1PR1
2	T_H1_	11.2	18.0	30.6	CCL5, GZMK, GZMA, CXCR3, NFKBIA, FOS, TNFAIP3, CD69, ZPF36L2, BTG1, ZPF36, CXCR4, IL7R, ITGAL, ITGB2
3	T_H17_	12.2	11.2	2.5	LTB, KLRB1, CCR6, RORC, AQP3, USP10, NME2, YWHAH, TIMP1, IL7R
4	T_PD‐1_	6.5	4.4	16.6	CST7, NKG7, GZMH, PRF1, GZMK, GZMA, PDCD1, ITGAL, ITGB2, S1PR5, TBX21
5	_MUC_T_M_	8.9	5.8	1.5	CRIP1, TRADD, LIMS1, ITGA4, UCP2, CORO1B, CD74, ITGB7, TCF7, S1PR4, FCMR
6	_REG_T_M_	7.2	5.4	6.9	FOXP3, GBP5, TNFRDF1B, FCMR, IL10RA, CD74, CD27, UCP2, CDKN1B, S1PR4
7	T_H17‐BM_	1.8	0.8	14.1	KLRB1, NFKBIA, FOS, DUSP1, JUN, RORA, CD69, ZFP36L2, CD40LG, ZPF36, YWHAH, IL2, IL7R, RORC, CCR6
8	T_FH‐BM_	1.3	0.5	16.4	BTG1, TCF7, CD69, ZFP36, ZFP36L2, CD40LG, RESF1, IL2,
9	T_SLEEP_	0.4	0.6	1.7	BTG1, BTG2, ZFP36, ZFP36L2, SLFN5, IL10RA, RESF1, AHNAK, KMT2A, DDX17, SYNE2, ITGAL, ITGA4, ITGB2
10	T_MX_	0.3	0.3	0.2	IFI44L, OAS1, MX1, IFI6, ISG15, MX2, IRF7
11	T_HMOX1_	0.0	0.2	0.0	C1QB, HMOX1, APOE, APOC1, C1QA, CST3, SELENOP
		100.0	100	100	

*Note*: Functional annotation of TCR^+^ CD4^+^ memory T cells clusters. The signature genes used for functional annotation were manually selected from the resulting unbiased differential gene expression analysis at min pct 0.5 and 0.1 and log fold change threshold of 0.3 and 0.25, respectively. The entire differential expressed gene list and the significance of each are available as Supporting Information Data S1a.

**TABLE 2:** TCR^+^ CD8^+^ memory T cell gene expression signatures and frequencies.

CD8 Cluster	Annotation	Blood CD69^−^	BM CD69^−^	BM CD69^+^	Signature genes
0	T_CM_	18.7	36.0	13.6	FOXO1, BTG1, GZMK, JUNB, CXCR4, LYAR, TCF7, SELL, CCR7, IL7R, IL10RA, ITGA4, KLF2, DUSP2, CD27, ZFP36L2, FCMR, GPR183, FOS, FOSB
1	T_EM_	22.9	13.8	14.8	KLRB1, CEBPD, IL7R, AQP3, NFKBIA, SLC4A10, RORC, IL23R, CD40LG, RORA, S1PR5, PRF1, LTB, CD7, TNFAIP3, CD69, BTG2
2	T_PD‐1/GMZ_	12.0	6.3	39.4	CD69, CCL4, GZMA, CST7, GZMH, GZMK, CD74, NKG7, ITGB2, CMC1, ACTG1, CLIC1, SRSF7, CD2, KLF6, ITGAL, EOMES, PDCD1, TOX, CXCR4
3	T_HLA‐DR/TIGIT_	11.8	9.3	14.1	IRF1, HLA‐DRA, HLA‐DRB1, HLA‐DRB5, LIMS1, COTL1, TOX, TIGIT, PECAM1, TNFRSF9, NKG7, GMZK, PFN1, CORO1A, EOMES, PDCD1, BTG3, IL21R
4	T_C2_	16.3	12.5	1.4	SELL, CCR7, TCF7, S1PR1, FOXO1, GATA3, LEF1, NOSIP, ITGB1, LTB, LDHB, CD40LG, IL6R
5	T_C1_	10.7	6.3	0.4	S1PR1, S1PR4, S1PR5, TBX21, GZMH, CST7, TMEM123, CX3CR1, ZEB2, CD52, IFITM2, LGALS1, KLF2, ANXA1, TGFB1, CD63, ZNF683
6	T_HOBIT/KLF6_	5.8	8.3	4.1	ZNF683, CCR7, TCF7, FOXO1, IL7R, PLP2, PTGER2, CD52, FOS, IFITM1, ITGA4, KLF6, TAGLN2, GAS5, GPR171, BCL2
7	T_PD1‐BM_	0.0	3.6	5.7	EOMES, SAT1, FTL, FTH1, C1QA, C1QB, C1QC, CD74, ID2, IKZF3, FOS, FOSB, ZFP36L2, SRSF7, GNAI2, KLF6, IL21R, PDCD1, TOX, CXCR6, CXCR3, FOXO3
8	T_PD4‐BM_	0.9	2.0	4.3	EOMES, CST7, ZFP36, DUSP2, CCL4, IL2RB, CD74, NKG7, GNAS, GZMK, IL16, ZFP36L1, PDCD4, STAT5A, CCL3, CXCR6
9	T_Q‐BM_	0.4	0.9	1.7	CD69, IKZF3, GNA13, JUN, SYNE1, ETS1, IL10RA, BTG1, TOX, TIGIT, NFKB1, IL16, ZFP36, CCR4, FOSB, SLFN5
10	T_HB_	0.1	0.8	0.6	HBA1, HBA2, HBB, PYHIN1, CCL4, GZMK, CLIC1, CMC1, SHISA5, SIT1
11	T_CYC_	0.3	0.2	0.0	MKI67, CDK6, CDK4, CDK1, CDK2
		100.0	100.0	100.0	

*Note*: Functional annotation of TCR^+^ CD8^+^ memory T cells clusters. The signature genes used for functional annotation were manually selected from the resulting unbiased differential gene expression analysis at min pct 0.5 and 0.1 and log fold change threshold of 0.3 and 0.25, respectively. The entire differential expressed gene list and the significance of each are available as Supporting Information Data S1b.

**Cluster 0** of CD8^+^ memory T cells consisted of SELL and CCR7 expressing central memory cells (T_CM_) (Figure 1I, Table 2). 36% of CD69^−^ T_BM_ belonged to this cluster, as compared with 19% of T_BL_ and 14% of CD69^+^ T_BM_. Cells in **cluster 1** expressed KLRB1 and PRF1 but not SELL, identifying them as effector memory cells (T_EM_). In contrast to CD69^−^ T_BL_, CD69^−^ T_BM_ in cluster 1 upregulated expression of the CD69 gene, although they did not express it on the surface (Figure S1C, see also Figure 3B; Figure S3C,D). CD8^+^ T_M_ of **cluster 2** resembled T_PD‐1_ CD4^+^ Tm in that they expressed granzyme genes, PD‐1, and EOMES. They also transcribed the CD69 gene, but surface CD69^−^ T_BM_ did not express it on the cell surface and expressed the transcription factor Krüppel‐like factor 2 (KLF2) (Figures S1C and S3C,D). We designated them as CD8 T_PD‐1/GMZ_. About 40% of the CD8^+^ T_BM_ belonged to this cluster. They correspond to EOMES^+^T_CM_ and T_EM_ expressing mainly GzmK in the accompanying paper of Pulvirenti et al. [25]. Cells in **cluster 3** expressed HLA‐DR, HLA‐DRB1, HLA‐DRB5, TOX, and TIGIT. We designated them as T_HLA‐DR/TIGIT._Cells of **cluster 4** expressed SELL, CCR7, GATA3, and IL6R, which classifies them as T_C2_ memory cells [24, 33, 34]. The signature genes of **cluster 5** included TBX21, GZMH, and ZNF683 (HOBIT), identifying them as T_C1_. They also expressed KLF2 and its target genes, including S1P receptor genes [9, 35, 36]. T_C1_ and T_C2_ cells were essentially absent from CD69^+^ T_BM_. Their S1PR1‐negative counterpart were cells in **cluster 6,** expressing KLF6, but not KLF2, and ZNF683 (HOBIT). We named them T_HOBIT/KLF6_ CD8^+^ memory T cells. Cells in **cluster 7** were designated as T_PD1‐BM_. We named **cluster 8** T_PD4‐BM_. Cells in **cluster 9** (T_Q‐BM_) expressed BTG1, SLF5, and ZFP36, indicating their quiescence. “Cells” in **cluster 10** were probably doublets of T cells and red blood cells since they expressed HBA1, HBA2, and HBB. Cells of **cluster 11** expressed MKI67 and CDKs, that is, were proliferating.

In summary, CD69^+^ CD4^+^ T_BM_ were essentially composed of cells in clusters 2, 4, 6, 7, and 8 (T_H1_, T_PD‐1, REG_T_M_, T_H17‐BM_, and T_FH‐BM_). Some cells of clusters 2, 4, and 6 were also found among CD69^−^ T_BL_ and T_BM_. CD69^+^ CD8^+^ T_BM_ were more diverse, containing cells of clusters 0, 1, 2, 3, 6, 7, and 8 (T_CM_, T_EM_, T_PD‐1 /GZM_, T_PD‐1,_ T_HLA‐DR/TIGIT_, T_HOBIT/KLF6_, T_PD‐4_). Cells of all these clusters, except clusters 7 and 8, were also found among CD69^−^ T_BL_ and T_PD4‐BM_. Cells of CD8 cluster 2 were significantly enriched among CD69^+^ T_BM_.

### 2.2 T Cell Antigen Receptor Repertoires Indicate Compartmentalization

**FIGURE 2:**
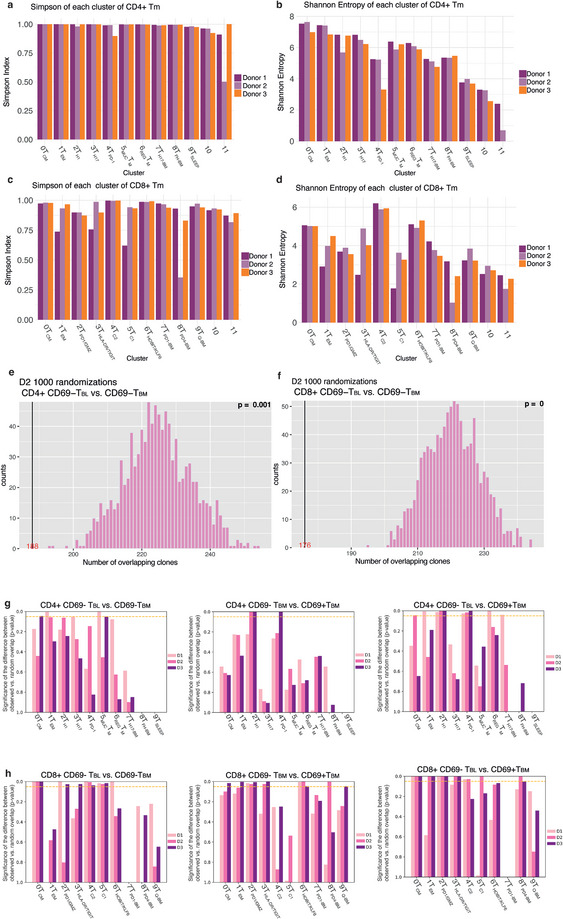
T cell receptor repertoires. The clonotype diversity of TCR^+^ (A) CD4^+^ and (C) CD8^+^ memory T cells is indicated by the Simpson index calculated for each cluster and donor. Likewise, the Shannon Entropy Index of (B) CD4^+^ and (D) CD8^+^ memory T cells of each cluster by donor. Histograms comparing the number of observed overlapping clonotypes to the overlap of (1000×) random reshuffling of TCR clonotypes for CD69^−^ T_BL_ and CD69^−^ T_BM_ of (E) CD4^+^ and (F) CD8^+^ memory T cells. A black line and a red number indicate the observed overlap. The bar histogram indicates the random overlaps. The *p*‐value indicates the probability that observed and random overlap are the same. Histograms are shown for one representative donor each. (G, H) Probabilities represented as *p*‐values on the *y*‐axis of TCR repertoire overlaps comparing observed versus random overlap for each cluster and donor. The dotted yellow line indicates a *p*‐value of 0.05.

We compared the T cell receptor (TCR) repertoires of T_BL_, CD69^+^, and CD69^−^ T_BM_ to investigate whether they represent distinct compartments of immunological memory. The overall diversity of the TCR repertoires for each donor is indicated by the Simpson Index [37] and the Shannon Entropy index [38] for each cluster (Figure 2A–D). The Simpson indices reflect the probability that any two TCR from two cells in a cluster are different. They are consistently at about 1 for all clusters of CD4^+^ T_BL_ and T_BM_ (Figure 2A). For CD8^+^ T_BL_ and T_BM_, the Simpson indices range from 0.25 to 1, indicating less diversity (Figure 2B). The Shannon Entropy indices, which account for clone sizes in addition, show a wide range of diversity of the cells, particularly for CD4^+^ memory T cells (Figure 2B,D; Figure S4).

To determine whether two populations of memory T cells share their TCR repertoires or not, we performed random shuffling of the observed TCRs of those populations. We then compared the distribution of shuffled random overlaps with the observed experimental overlap (Figure 2E,F; Figure S2) [39]. If the observed overlap is significantly lower than the randomized overlaps, it argues for distinct repertoires and the two populations making up distinct memory compartments. If observed and randomized overlaps do not differ, the two populations could either represent one compartment at two locations or two distinct compartments but derived from the same original immune reactions.

The TCR repertoires of all CD4^+^ T_BL_ and T_BM_, including both CD69^−^ and CD69^+^ subsets, are significantly different in donors 1 and 2, as are the TCR repertoires of CD8^+^ memory T cells in all donors (Figure 2E,F; Figure S2).

To understand whether the compartmentalization of the TCR repertoires is specific to each original activation imprint, we also compared repertoires cluster by cluster. Of the repertoires of CD4^+^CD69^−^ T_BL_ and T_BM,_ in at least one donor, T_CM_, T_EM_, and _MUC_T_M_ (clusters 0, 1, 5) were significantly different (Figure 2G; Table S3). When comparing CD69^+^ and CD69^−^ T_BM_, we found that T_H1_ and T_PD‐1_ (clusters 2 and 4) were significantly different in one and two donors, respectively. (Figure 2G; Table S3). Comparing CD69^+^ T_BM_ to CD69^−^ T_BL_ we observed that T_CM_ (cluster 0) from donor 2, T_EM_, _REG_T_M_, and T_H17‐BM_ (clusters 1, 6, 7) from donor 1 andT_H1_ and T_PD‐1_ (clusters 2 and 4) from all donors, had significantly different repertoires (Figure 2G; Figure 2; Table S3).

For CD8^+^ memory T cells, comparing T_BL_ to CD69^−^ T_BM_, T_CM_, T_C2_, and T_C1_ (clusters 0, 4, and 5) of all donors had significantly different repertoires. T_PD‐1/GZM_ (cluster 2) was significantly different for donors 1 and 3, and T_HOBIT/KLF6_ (cluster 6) of donor 1 (Figure 2H). Comparing the repertoires of CD8^+^CD69^+^ and CD69^−^ T_BM_, T_CM_ (cluster 0) of donor 1, T_EM_ (cluster 1) of donor 3, T_HLA‐DR/TIGIT_ (cluster 3) of donors 2 and 3, T_PD‐4‐BM_ (cluster 8) of donor 2 and T_Q‐BM_ (cluster 9) of donor 3, differed significantly. The repertoires of T_PD‐1/GZM_ and T_HOBIT/KLF6_ (clusters 2 and 6) were significantly different for all three donors (Figure 2H; Table S3). For T_BL_ and CD69^+^ T_BM_, repertoires were significantly different for T_CM_ and T_PD‐1/GZM_ (clusters 0 and 2) in all three donors, for T_EM_ and T_HLA‐DR/TIGIT_ (clusters 1 and 3) of two donors, T_C1_ and T_PD4‐BM_ (clusters 5 and 8) of one donor (Figure 2H; Table S3).

In summary, comparing the TCR repertoires of memory T cells present in blood and bone marrow indicates that cells from the same cluster have different TCR repertoires, at least in one donor each, and thus comprise distinct compartments of memory.

### 2.3 Specific Transcriptional Signature Genes of Surface CD69^−^ T_BM_

**FIGURE 3:**
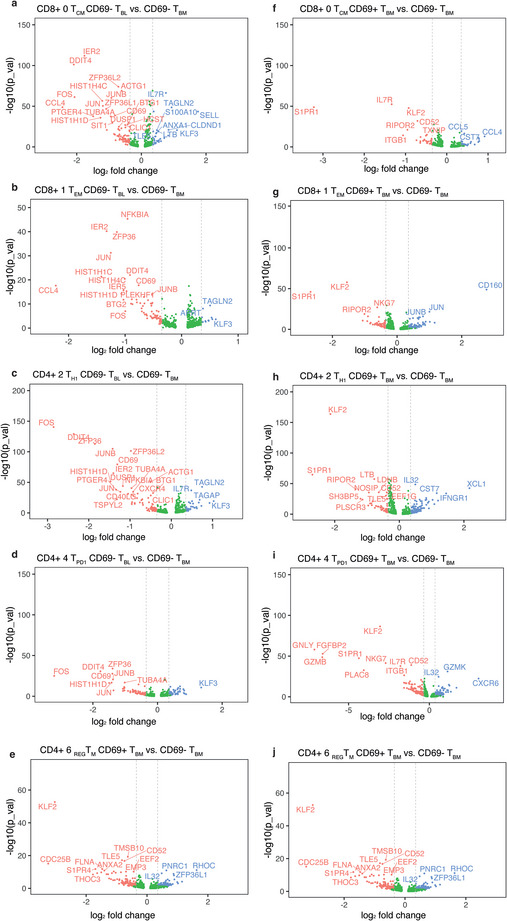
Tissue‐specific, cluster‐specific gene expression. (A–J) Pairwise comparison of gene expression of cells that clustered together but were isolated from a given tissue, as indicated above each panel. Panel labels show CD4^+^ or CD8^+^ cells, the cluster number with its functional annotation followed by the origin of the cells used for the differential gene expression analysis CD69^+^ or CD69^−^ and T_BL_ or T_BM_. The *x*‐axis is the log2‐fold‐difference in gene expression versus the −log10 of the *p*‐values (*y*‐axis). Significantly differentially expressed genes (Wilcoxon rank sum test *p* < 0.05 and log2FC > 0.35) are labelled in blue and red. Red labels are upregulated for CD69^–^ T_BM_ in all panels shown. The labels of T_BL_ are cells isolated from blood or T_BM_ for cells isolated from bone marrow (BM). To note, the first population mentioned in the panel is the reference for the given comparison, thus, blue/upregulated genes are expressed on these cells, while red/downregulated genes are expressed in the second population mentioned on each panel title.

While cells from the same cluster in blood and bone marrow share the expression of signature genes to some extent, the pairwise comparison of transcriptomes of cells from the same clusters in blood and bone marrow also identified specific transcriptional differences (Figure 3). For clusters 2, 4, 6 of CD4^+^ and clusters 0, 1 of CD8^+^ memory T cells, T_BM_ differed from T_BL_ by expressing genes like ZFP36, ZFP36L2, ZFP36L1, ACTG1, and BTG1 genes which indicate repression of cytokine translation and induction of quiescence [27, 28, 31, 40, 41]. They also differentially expressed FOS, JUN, and JUNB, that is, components of the transcription factor AP‐1. On the other hand, T_BL_ had differentially upregulated expression of the genes IL7R, TAGLN2, ANXA1, SELL, KLF3, TLE5, and LTB, which are functionally connected to mobility and navigation [42, 43, 44]. Interestingly, surface CD69^−^ T_BM_ of all clusters analyzed also transcribed the CD69 gene (Figure 3A,E) and expressed intracellular CD69 protein (Figure S6), despite their lack of expression of CD69 on the cell surface (Figures S1C and 3A–D). For all clusters analyzed, CD69^−^ T_BL_ show upregulation of KLF3, a gene related to quiescence [45]. Only a few genes were differentially expressed between CD69^−^ and CD69^+^ T_BM_ of CD8 clusters 0 and 1 and of CD4 clusters 2, 4, and 6. Remarkably, they did not differ significantly in the transcription of CD69. However, they did differ in the transcription of KLF2 and its target S1PR1 (Figure 3E–H). Notably, it has been described that CD69 and S1PR1 dimerize and are internalized by cells expressing both genes [14].

In summary, comparison of the transcriptomes of circulating T_BL_ and surface CD69^−^ and CD69^+^ T_BM_ of CD8 clusters 0 and 1, and of CD4 clusters 2, 4 and 6, revealed that surface CD69‐negative T_BM_ transcribed the CD69 gene, expressed genes of quiescence, like BTG1, and the transcription factor AP‐1. They differed from CD69^+^ T_BM_ by expression of KLF2 and S1PR1, but both transcribed CD69 to the same degree.

## 3 Discussion

The bone marrow has been identified as the home of memory T cells conferring long‐term memory to systemic antigens [1, 3]. Tissue‐resident memory T cells in barrier tissues like skin, intestine and lung express CD103 and CD69 [8, 9]. Memory T cells of the bone marrow do not significantly express CD103, but about 30% of the CD4 and 60% of the CD8 memory T cells of human bone marrow do express CD69 on the cell surface [3]. Those cells also express the key signature genes that have been associated with tissue‐resident memory T cells in barrier tissues [6]. The nature of CD69^−^ memory T cells in human bone marrow has so far been elusive. Here, we have analyzed the transcriptomes and TCR repertoires of individual resting CD4 and CD8 memory T cells from paired blood and bone marrow samples from three human donors. According to their transcriptomes, memory CD4 and CD8 T cells could be separated into 10 clusters each. Their signature genes reflect differential instruction during activation, for example, central versus effector memory, T_H1_ versus T_H17_. They also reflect their lifestyle in the phase of resting memory and mobility for memory T cells circulating in blood and quiescence for cells resting in the bone marrow. Cells of most clusters are present both in blood and bone marrow but have different TCR repertoires, at least in one of the donors analyzed. This indicates that they belong to different compartments of memory and suggests that the memory T cells of the bone marrow are residents of the bone marrow. It should be noted that the clusters that do not show a clearly distinct TCR repertoire could also belong to different compartments in the memory phase of immunity; it is just not evident from their repertoires at the sample sizes analyzed here.

Specific differences in gene expression between cells of the same cluster in blood versus bone marrow provide an interesting clue as to how the CD69^−^ T_BM_ may be maintained in the bone marrow. Both CD4 and CD8 T_BM_ of a given cluster, not expressing CD69 on the cell surface (CD69^−^), are actually transcribing the CD69 gene and expressing it intracellularly. And unlike their CD69^+^ cousins of the bone marrow, they also transcribe the S1PR1 gene and the gene encoding the transcription factor KLF2. While CD69 is required for memory T cells to enter the bone marrow [46, 47, 48], its relevance for the maintenance of memory T cells there has not been shown. It could be speculated that CD69 intracellular expression of surface CD69^−^ T_BM_ prevents expression of the receptor for sphingosine‐1‐phosphate 1 (S1PR1) on the cell surface, blindfolding the cells for perception of the egress signal sphingosine‐1‐phosphate. This would be similar to the situation in germinal centers during an immune reaction [49], where CD69 is reported to dimerize with S1PR1 and promote internalization of the dimer, rendering the cells negative for expression of CD69 and S1PR1 on the cell surface [14, 15]. Expression of both genes, CD69 and S1PR1, and the S1PR1 inducing transcription factor KLF2, supports the notion that the surface CD69^−^ T_BM_ are residents of the bone marrow, and not just a detachment of circulating memory T cells. The evidence for both bone marrow CD69^+^ and CD69^−^ memory T cells, as residents in the bone marrow, argues that we could designate them both as the T_BM_ compartment of immunological memory. The differential expression of KLF2 by surface CD69^−^ resident T_BM_ may also reflect a different instruction and maintenance of the quiescent memory state [50] as compared with CD69^+^ T_BM_. Overall, unraveling the heterogeneity of T_BL_ and T_BM_ at the single‐cell level discloses an unforeseen diversity. For example, only a fraction of the T_BM_ express HOBIT, a transcription factor claimed to be mandatory for tissue‐resident memory T cells [51]. Whether this diversity also implies different maintenance mechanisms remains to be shown. In contrast to T_BL_, T_BM_ is maintained in contact with stromal cells [1, 11] and may depend on survival signals from them, activating the PI3K/AKT pathway, in analogy to memory plasma cells of the bone marrow [52]. The upregulated expression of the AKT‐dependent transcription factor AP‐1 genes in both CD69^+^ and CD69^−^ T_BM_, as compared with circulating memory T cells, could be taken as an indication of this [29, 51].

In summary, here we present an in‐depth analysis of the transcriptional diversity of human memory T cells circulating in the blood (T_BL_) or residing in the bone marrow (T_BM_). The diversity observed reflects both differences in their original instruction and differences in their maintenance in the phase of resting memory. T_BL_ and surface CD69^−^ and CD69^+^ T_BM_ qualify as distinct compartments of memory, indicated by their different TCR repertoires. For surface CD69^−^ T_BM_, we show their transcription of both the CD69 and the S1PR1 genes, suggesting that they are maintained in bone marrow by CD69‐mediated internalization of S1PR1, blindfolding the cells for the egress signal S1P.

## 4 Data Limitations and Perspectives

To our knowledge, this is the first direct comparison of human resting CD4 and CD8 memory T cells from paired blood and bone marrow samples on the level of single cell transcriptomes, T cell receptor repertoires, and cell surface immunophenotyping.

Since the samples were obtained in the context of surgical joint replacement, they were limited in size, and each compartment analyzed per patient contained only between 835 and 4500 cells each. More cells would have allowed us to also analyze small clusters in more detail and would have eased the repertoire comparisons, in particular for the more diverse CD4 memory cells with their high Simpson indices. Due to limitations in experimental options to analyze the human immune system, and in particular the bone marrow in vivo, our conclusions here are based on differences and similarities in gene expression signatures and on comparisons of TCR repertoires between memory T cells of blood and bone marrow. These comparisons provide convincing evidence that memory T cells of blood and bone marrow belong to different compartments and indicate that memory T cells of bone marrow are residents of the bone marrow. How stable this residency is and under which circumstances such cells are mobilized remain less clear [2].

The source of samples also points to a second limitation, in that they all were derived from elderly patients and may differ from those of young humans. It also remains to be shown whether the underlying osteoarthritic degeneration of the joint or the surgical intervention may have impacted on the diversity of memory T cells, for example, the proliferating CD8^+^ cluster 11 cells. On the other hand, it is reassuring that all three donors showed all transcriptional clusters at comparable frequencies despite their obviously different lifelong immunological experiences and genetic backgrounds.

## 5 Materials and Methods

### 5.1 Human Donors

The recruitment of human donors was done in accordance with the Ethics Committee of the Charité‐Universitätsmedizin Berlin, in compliance with the Declaration of Helsinki (EA1/105/09 and EA1/261/09). For all donors included in the study, informed consent was obtained for the sampling of blood and bone marrow. No compensation was provided for this. Bone marrow and peripheral blood were obtained from three donor subjects undergoing total hip arthroplasty without any underlying malignancy or rheumatic or autoimmune diseases (2 females, 1 male, with a median age of 71 years, see Table S1).

### 5.2 Blood and Bone Marrow T Cell Isolation

Bone marrow samples were transferred to a 50 mL tube and vortexed to loosen the cells from bone fragments. Afterwards, we rinsed them with PBS/1% BSA/5 mM EDTA/2 µg/mL Actinomycin D to obtain a cell suspension and disrupt de novo transcription and preserve in vivo transcriptomes [53]. The blood collected in EDTA vacutainer tubes was transferred to a 50 mL tube. Then 50µL per 1 mL of sample of CD3^+^ Microbeads (cat.130‐090‐874, Miltenyi Biotec) was added to the bone marrow and blood samples. We proceeded according to the manufacturer's instructions, with a modified buffer containing Actinomycin D at 2 µg/mL.

Enriched cells were incubated with FcR Blocking Reagent (cat. 130‐059‐901, Miltenyi Biotec) following manufacturer's instructions and subsequently stained for 30 min at 4°C with the following human antibodies: CD8‐FITC (clone GN11/134D7, in‐house, 1:1000), CD45RO‐PE (clone UCHL1, in‐house, 1:200), and CD4‐BV650 (clone OKT4, BioLegend, cat. 317436, 1:50). Simultaneously, cells were incubated with DNA barcoded antibodies for Cellular Indexing of Transcriptomes and Epitopes by Sequencing (CITE‐seq, see antibody list). DAPI was added before sorting at 1:100 dilution to allow dead cell exclusion.

For immunofluorescence experiments, bone marrow mononuclear cells from three donors were isolated by density gradient centrifugation after fragmentation of the tissue as reported previously by our group [2].

### 5.3 Single Cell Suspension Sorting

Cells were sorted using an MA900 Multi‐Application Cell Sorter (Sony Biotechnology). Viable CD4^+^ CD45RO^+^ and CD8^+^CD45RO^+^ T cells were recovered in Eppendorf tubes. The gating strategy is shown in Figure S1B. Sorted cells were counted using a MACSQuant Analyzer 16 (Miltentyi Biotec). The cells were further processed for single‐cell RNA sequencing.

### 5.4 Single Cell Library Preparation and Sequencing

Single‐cell RNA library construction and sequencing were done as previously described [35]. Chromium Next GEM Single Cell 5’ reagent kits v2 (dual index) with featured barcode technology for cell surface protein (CITE) mapping (10X Genomics) were used as stated in the manufacturer's protocol. Final CITE‐Seq libraries were generated after index PCR with dual Index Kit TN Set A (10X Genomics), while final GEX and TCR libraries were generated after fragmentation, adapter ligation, and final index PCR with a dual Index Kit TT Set A (10X Genomics). Libraries were quantified using a Qubit HS DNA assay kit (LifeTechnologies), and fragment sizes were determined using an HS NGS Fragment (1–6000 bp) kit (Agilent). All libraries (GEX and CITE and TCR libraries) were sequenced on a NextSeq2000 sequencer (Illumina) using a P3 reagent kit (100 cycles) (Illumina) and following the sequencing conditions recommended by 10x Genomics: read1: 26 nt, read2: 90 nt, index1: 10 nt, index2: 10 nt [35].

### 5.5 Single Cell Transcriptome Analysis

Raw sequence reads from the GEX and CITE libraries were processed using cellranger (version 5.0.0). Demultiplexing, mapping, and detection of intact cells, as well as quantification of gene expression, was performed using cellranger's count pipeline in default parameter settings with refdata‐gex‐GRCh38‐2020‐A as a reference and an expected number of 3000 cells per sample. GEX‐libraries were merged without size normalization and reanalyzed without genes related to TCRs, that is, with related biotype in the reference GTF‐file using the aggr and the reanalyze pipeline [35]. Raw sequence reads from the TCR libraries were processed using the cellranger's vdj pipeline in default parameter settings with refdata‐cellranger‐vdj‐GRCh38‐alts‐ensembl‐2.0.0 as reference and merged without size normalization with cellranger's count.

Further analysis was conducted in R (version 4.2.3) utilizing the Seurat package (version 5.0.1). A Seurat object was created from the merged, reanalyzed transcriptome profiles from CellRanger using the Read10x and CreateSeuratObject function. The data were log‐normalized using the NormalizeData function with a scale factor of 10,000. T‐cell receptor (TCR) information derived from the filter_contig_annotation.csv file from TCR sequencing was incorporated based on identical cellular barcodes. Clones were defined by sharing the exact same combination of alpha and beta CD3R aa sequence. Cells lacking complete annotations for both the alpha and beta chains of the TCR were excluded from subsequent analyses. Manual gating on the log‐transformed CITE‐seq counts for CD4, CD8, and CD69 was performed to define six T cell subsets: Blood CD4^+^, BM CD4^+^ CD69^+^, BM CD4^+^ CD69^−^, Blood CD8^+^, BM CD8^+^ CD69^+^, and BM CD8^+^ CD69. CD4^+^ and CD8^+^ T cells were subset and independently harmonized to remove organ and donor‐specific differences. For harmonization, 2,000 variable features were defined using the FindVariableFeatures function with the vst as the selection method and scaled with the ScaleData function. A principal component analysis (PCA) was performed with RunPCA to compute 50 principal components and processed with RunHarmony (harmony R package) in default parameter settings [54]. Based on the harmony reduction, a PCA was executed to compute 50 principal components using RunPCA, followed by UMAP dimensionality reduction with RunUMAP using the first 50 dimensions. Transcriptionally similar clusters were identified through shared nearest neighbor (SNN) modularity optimization by FindNeighbors with harmony as reduction and the first 50 dimensions and following clustering with FindClusters at resolutions ranging from 0.1 to 1.0 in 0.1 increments [35]. Based on visual inspection of mitochondrial gene percentages, UMI counts, the number of identified genes, and the expression of typical marker genes projected onto the UMAP, we determined that clustering with a resolution of 0.6 for CD4^+^ memory T cells and 0.7 for CD8^+^ memory T cells best reflected the transcriptional community structure.

### 5.6 TCR Overlap Analysis

The statistical probability of overlap between groups was assessed by comparing the observed overlaps to expected overlaps in 1000 random shuffles of the group label, that is, keeping the size of the subsets constant. The global overlap between Blood CD4^+^, BM CD4^+^ CD69^+^, and BM CD4^+^ CD69^−^ was performed separately for CD4^+^ and CD8^+^ T cells and donors by shuffling the labels of two compared compartments at each time. For overlaps of specific subtypes, the probability of overlap was evaluated individually for each combination of two compartments, subtype, and donor. This approach was based on prior publications of our group [39].

### 5.7 Flow Cytometry

Viable bone marrow mononuclear cells of three additional donors, not listed in Table S1, were stained for surface CD69 and other markers, as indicated, with antibodies listed in Table S2. According to [55], for 15 min at 4°C. Cells were then washed and fixed with PBS 4% paraformaldehyde and washed again. For intracellular staining, cells were incubated with anti‐CD69‐Allophycocyanin‐Cyanin‐7 (FN50) in PBS with 0.05% Saponin (Sigma, S4521), or, as a technical control, in the absence of saponine for 10 min at room temperature. Cells were then washed and prepared for acquisition in MACSQuant Analyser 16 (Miltentyi Biotech).

## Author Contributions

Conceptualization: Andreas Radbruch, Emilia Schneider Revueltas, and Koji Tokoyoda. Methodology: Emilia Schneider Revueltas, Andreas Radbruch, Gabriela Maria Guerra, Marta Ferreira‐Gomes, Thomas Dörner, Hyun‐Dong Chang, and Anna Casanovas Subirana. Sampling: Carsten Perka, Simon Reinke, Sebastian Hardt, Christian Hipfl, and Thomas Dörner. Software: Emilia Schneider Revueltas, Pawel Durek, and Frederik Heinrich. Validation: Andreas Radbruch, Emilia Schneider Revueltas, Mir‐Farzin Mashreghi, Marta Ferreira‐Gomes, Hyun‐Dong Chang, and Pawel Durek. Formal Analysis: Emilia Schneider Revueltas and Andreas Radbruch. Investigation: Emilia Schneider Revueltas and Andreas Radbruch. Data Curation: Emilia Schneider Revueltas and Pawel Durek. Writing—original draft: Emilia Schneider Revueltas and Andreas Radbruch. Writing—review and editing: Andreas Radbruch, Emilia Schneider Revueltas, Marta Ferreira‐Gomes, and Jun Dong. Visualization: Emilia Schneider Revueltas. Supervision: Andreas Radbruch, Hyun‐Dong Chang, and Mir‐Farzin Mashreghi. Project administration: Andreas Radbruch, Emilia Schneider Revueltas, Gabriela Maria Guerra, and Ute Hoffmann. Funding acquisition: Andreas Radbruch, Jun Dong, Mir‐Farzin Mashreghi and Emilia Schneider Revueltas.

## Conflicts of Interest

The authors declare no conflicts of interest.

### Peer Review

The peer review history for this article is available at https://publons.com/publon/10.1002/eji.202451529.

## Supporting information



Supporting information

Supporting information

Supporting information

Supporting information

Supporting information

Supporting information

Supporting information

Supporting information

Supporting information

## Data Availability

Data used for this article were uploaded to the Gene Expression Omnibus (GEO) repository with accession codes GE278281 and GSE278283. Single‐nucleotide variants were removed from these files. Code availability: It is available under the following link: https://github.com/emilia‐srevueltas/scRNA_CD69n_memoryTcells. Other sections not detailed in the repository can be obtained upon request from the authors. The antibody list is provided in Table S2.
