# Supplementary material for: Surface CD69‐Negative CD4 and CD8 Bone Marrow‐Resident Human Memory T Cells
Source: Eur J Immunol. 2025 May 16;55(5):e202451529. doi: 10.1002/eji.202451529 (PMC12082384; doi:10.1002/eji.202451529)
Supplement: Supplementary file 6 — Supporting information [file EJI-55-e202451529-s002.pdf]

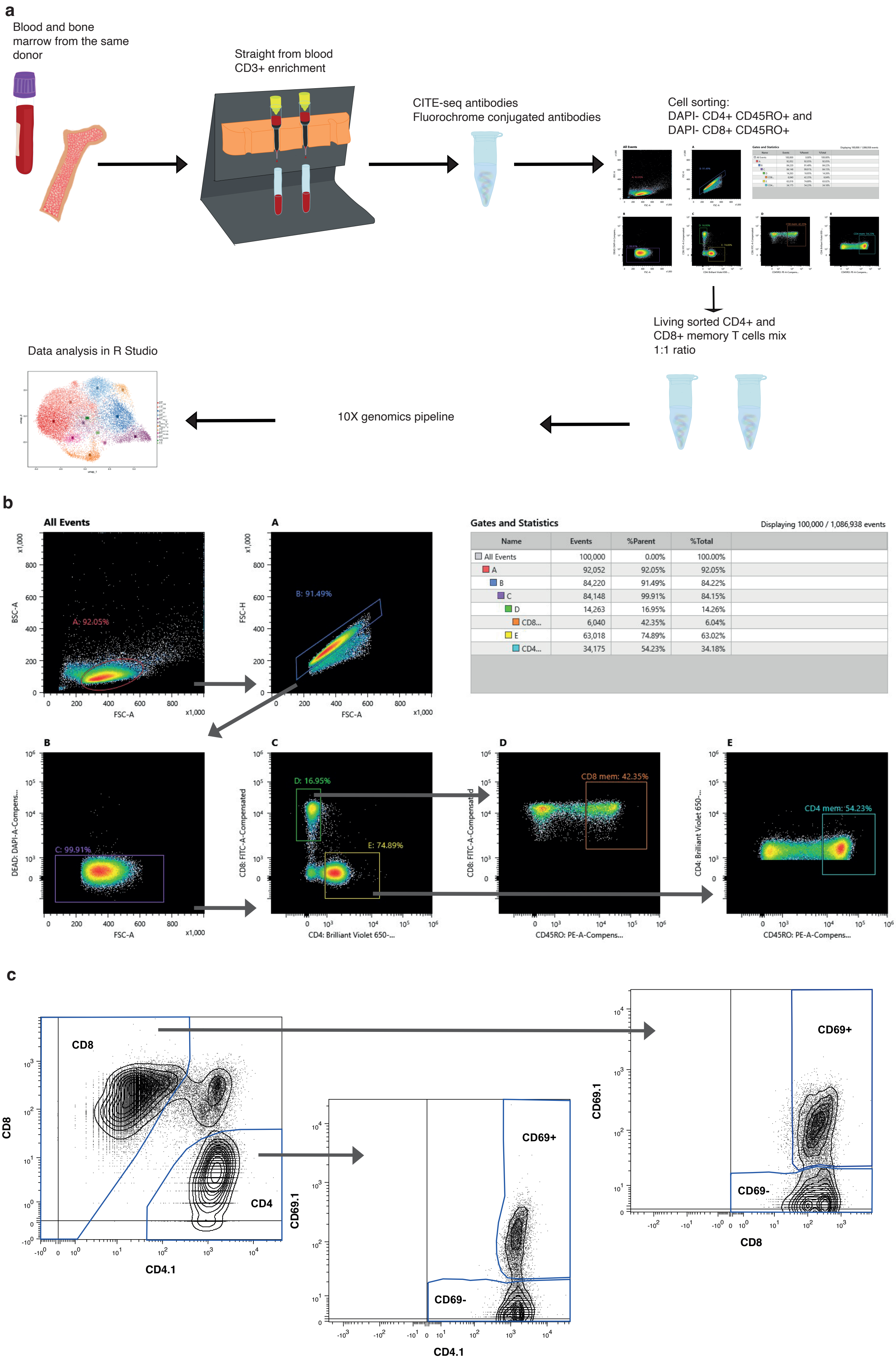

Supplementary Figure 1. Isolation and purification of memory T cells. (a) Visual scheme of the isolation process. (b) Gating strategy for fluorescence activated cell sorting on DAPI-/CD4+ or CD8+/CD45RO+(55). (c) Manual gating strategy based on sequencing data for annotation of (left) CD8+ or CD4+ followed by (center) CD4+/CD69+ or CD69- and (right) CD8+/CD69+ or CD69- populations for separation of the data for further analysis.

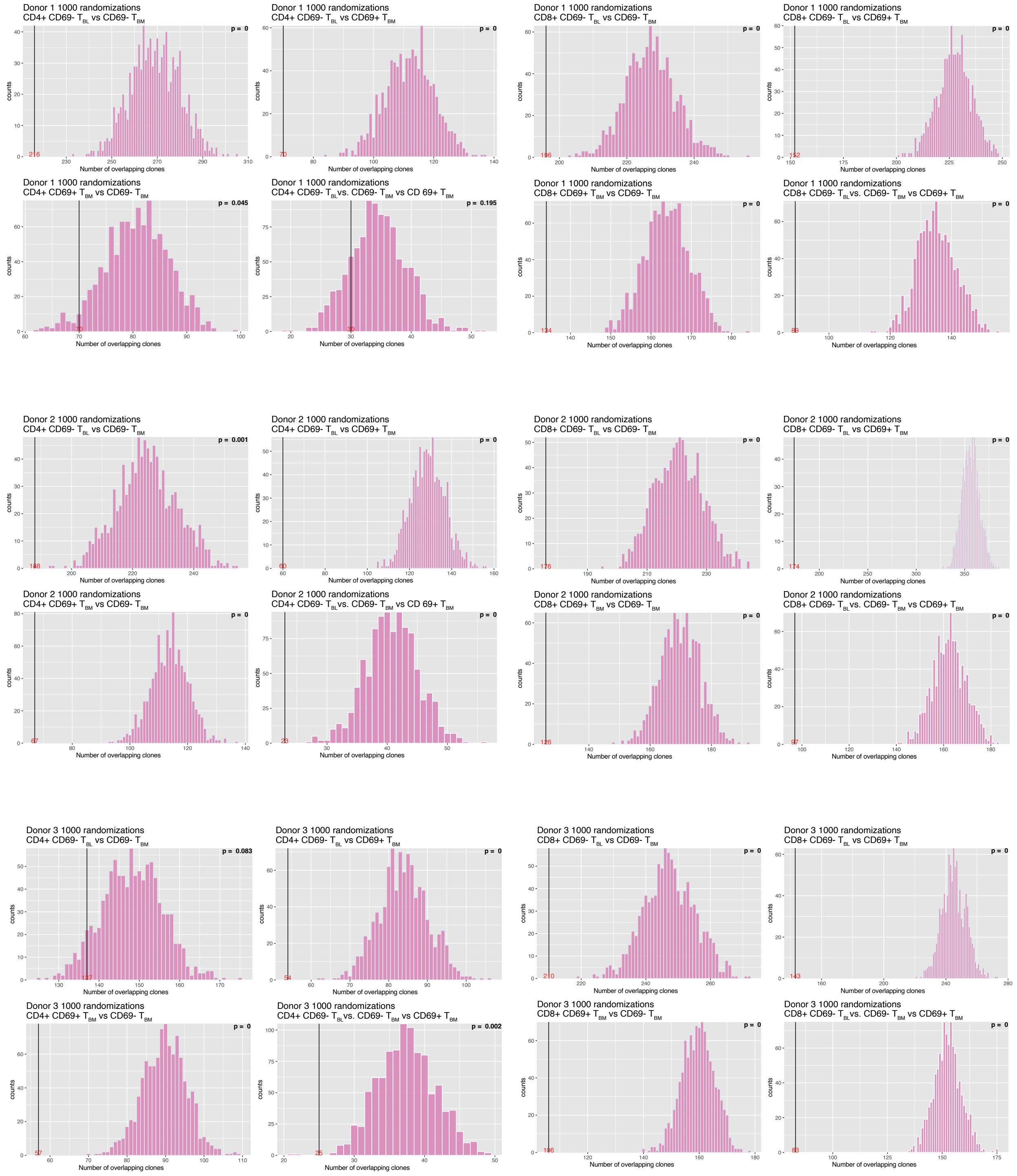

Supplementary Figure 2. Histograms comparing the number of observed overlapping clonotypes to the overlap of (1000x) random reshuffling of TCR clonotypes of all donors. Donor 1 upper panels, Donor 2 middle panels and Donor 3 lower panels. The populations analyzed are indicated on each plot. The observed overlap is indicated by a black line and red number. The bar histogram indicates the random overlaps. The p value indicates the probability that observed and random overlap are the same.

a

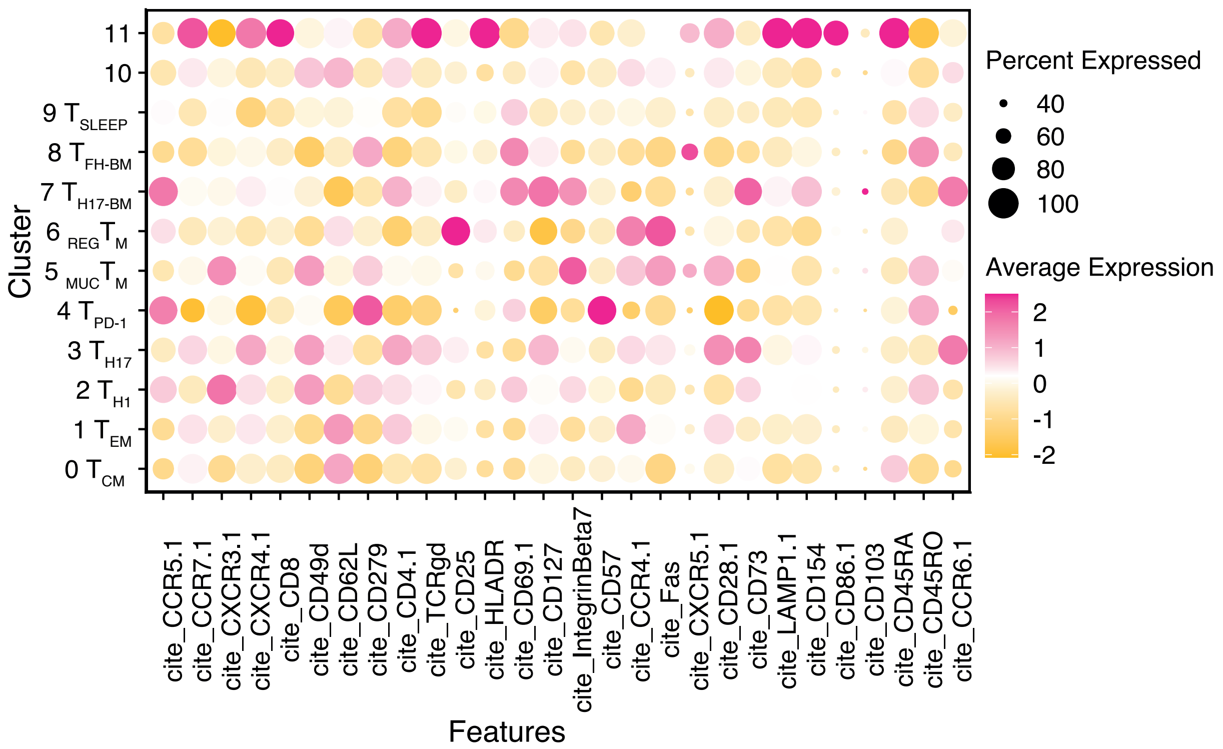

Cluster
11
10
9 T SLEEP
8 T FH-BM
7 T H17-BM
6 REG T M
5 MUC T M
4 T PD-1
3 T H17
2 T H1
1 T EM
0 T CM
cite_CCR5.1
cite_CCR7.1
cite_CXCR3.1
cite_CXCR4.1
cite_CD8
cite_CD49d
cite_CD62L
cite_CD279
cite_CD4.1
cite_TCRgd
cite_CD25
cite_HLADR
cite_CD69.1
cite_CD127
cite_IntegrinBeta7
cite_CD57
cite_CCR4.1
cite_Fas
cite_CXCR5.1
cite_CD28.1
cite_CD73
cite_LAMP1.1
cite_CD154
cite_CD86.1
cite_CD103
cite_CD45RA
cite_CD45RO
cite_CCR6.1
Features
Percent Expressed
40
60
80
100
Average Expression
2
1
0
-1
-2

b

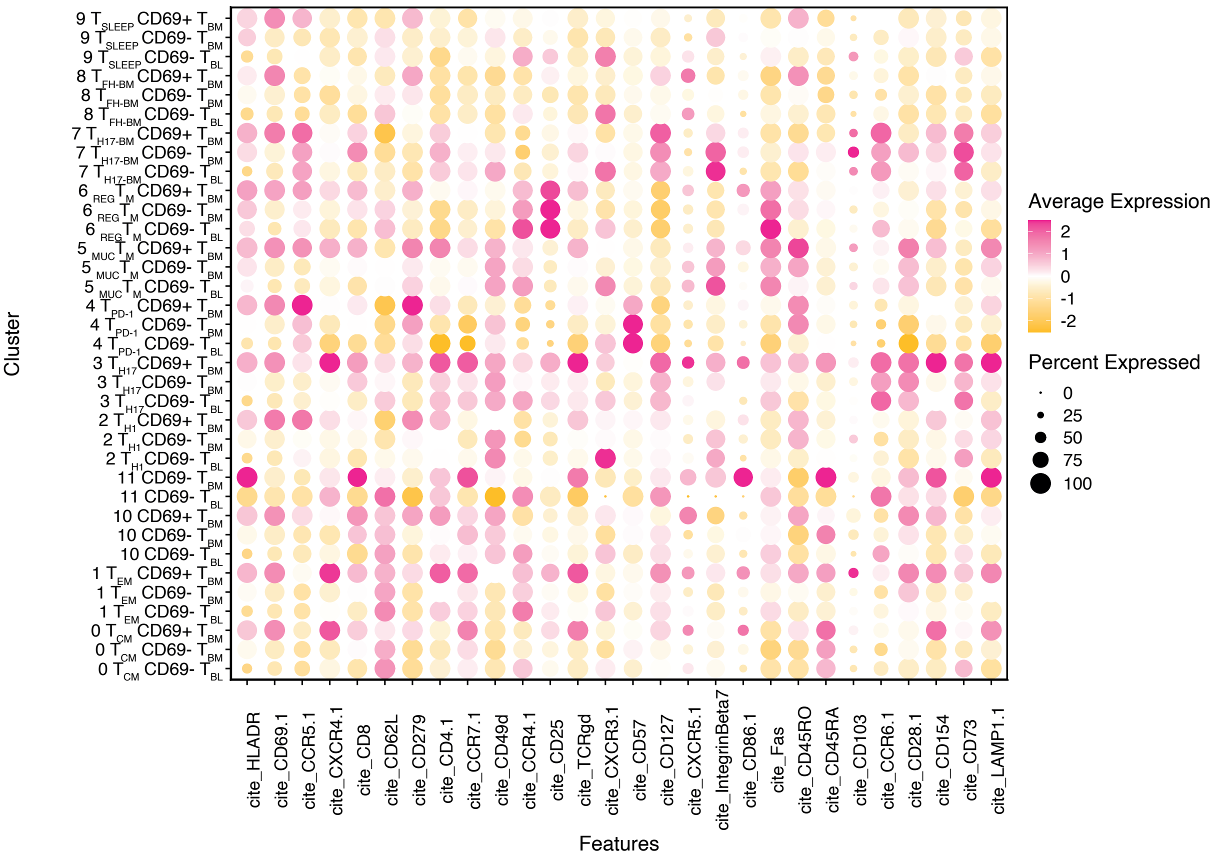

Cluster
9 T SLEEP CD69+ T BM
9 T SLEEP CD69- T BM
9 T SLEEP CD69- T BL
8 T FH-BM CD69+ T BM
8 T FH-BM CD69- T BM
8 T FH-BM CD69- T BL
7 T H17-BM CD69+ T BM
7 T H17-BM CD69- T BM
7 T H17-BM CD69- T BL
6 REG T M CD69+ T BM
6 REG T M CD69- T BM
6 REG T M CD69- T BL
5 MUC T M CD69+ T BM
5 MUC T M CD69- T BM
5 MUC T M CD69- T BL
4 T PD-1 CD69+ T BM
4 T PD-1 CD69- T BM
4 T PD-1 CD69- T BL
3 T H17 CD69+ T BM
3 T H17 CD69- T BM
3 T H17 CD69- T BL
2 T H1 CD69+ T BM
2 T H1 CD69- T BM
2 T H1 CD69- T BL
11 CD69- T BM
11 CD69- T BL
10 CD69+ T BM
10 CD69- T BM
10 CD69- T BL
1 T EM CD69+ T BM
1 T EM CD69- T BM
1 T EM CD69- T BL
0 T CM CD69+ T BM
0 T CM CD69- T BM
0 T CM CD69- T BL
cite_HLADR
cite_CD69.1
cite_CCR5.1
cite_CXCR4.1
cite_CD8
cite_CD62L
cite_CD279
cite_CD4.1
cite_CCR7.1
cite_CD49d
cite_CCR4.1
cite_CD25
cite_TCRgd
cite_CXCR3.1
cite_CD57
cite_CD127
cite_CXCR5.1
cite_IntegrinBeta7
cite_CD86.1
cite_Fas
cite_CD45RO
cite_CD45RA
cite_CD103
cite_CCR6.1
cite_CD28.1
cite_CD154
cite_CD73
cite_LAMP1.1
Features
Average Expression
2
1
0
-1
-2
Percent Expressed
0
25
50
75
100

c

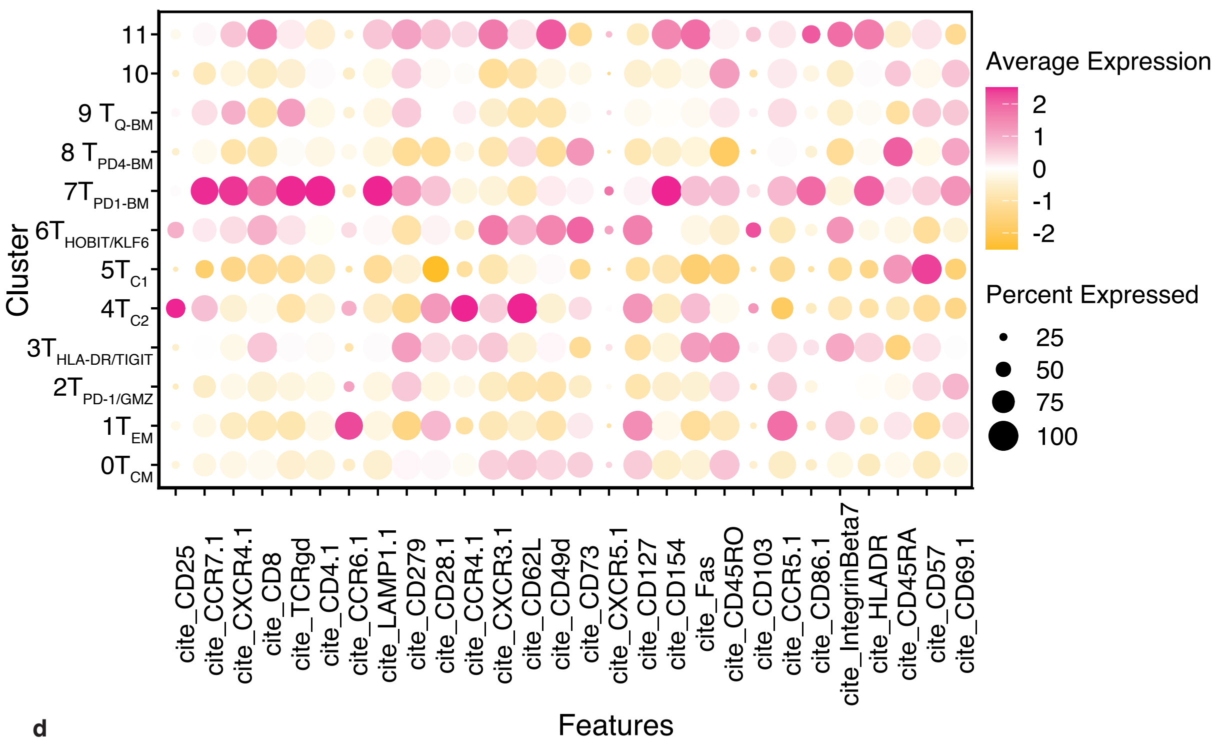

d

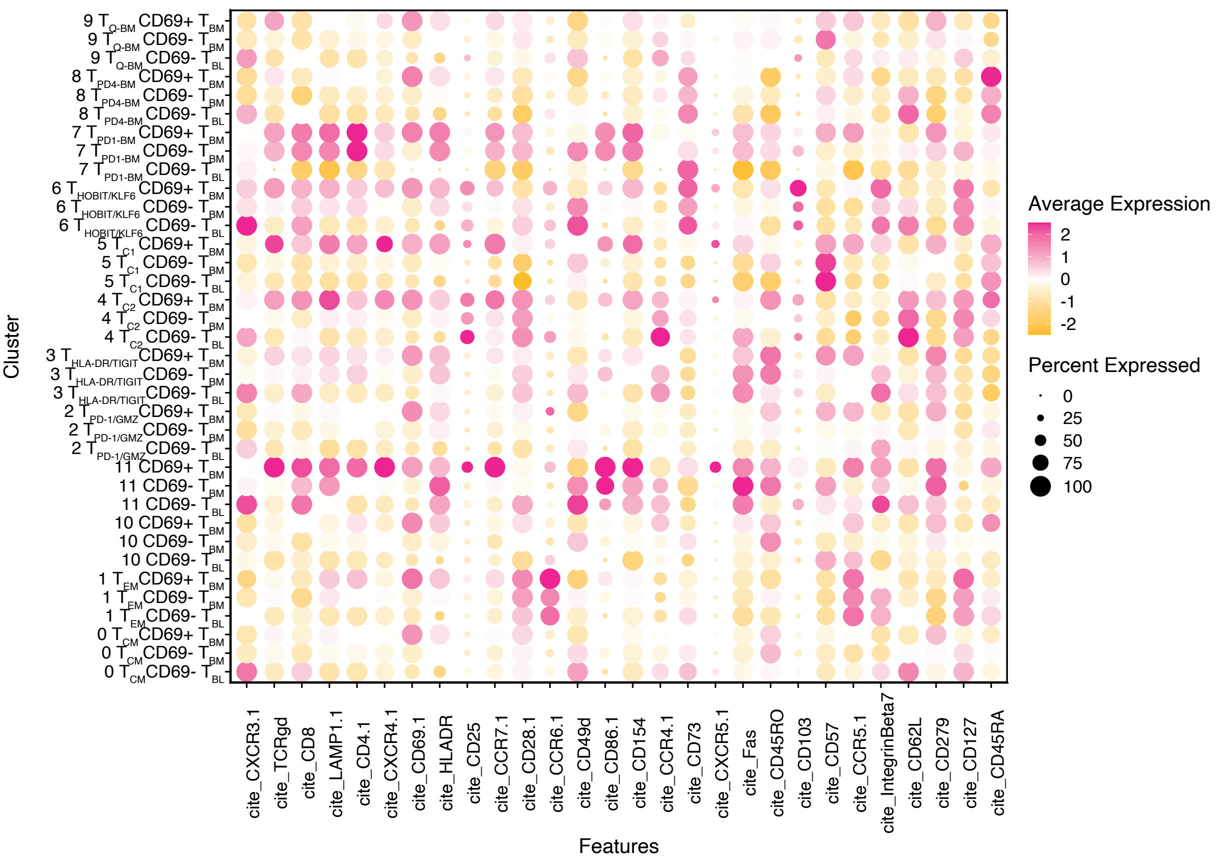

Supplementary Figure 3. CITE-seq antibody differential expression. (a) Differential expression of CITE-seq antibodies by cluster of CD4+ Tm. (b) Differential expression of CITE-seq antibodies by cluster and tissue of origin of CD4+ Tm. (c) Differential expression of CITE-seq antibodies by cluster of CD8+ Tm. (d) Differential expression of CITE-seq antibodies by cluster and tissue of origin of CD8+ Tm.

Supplementary Figure 4

a

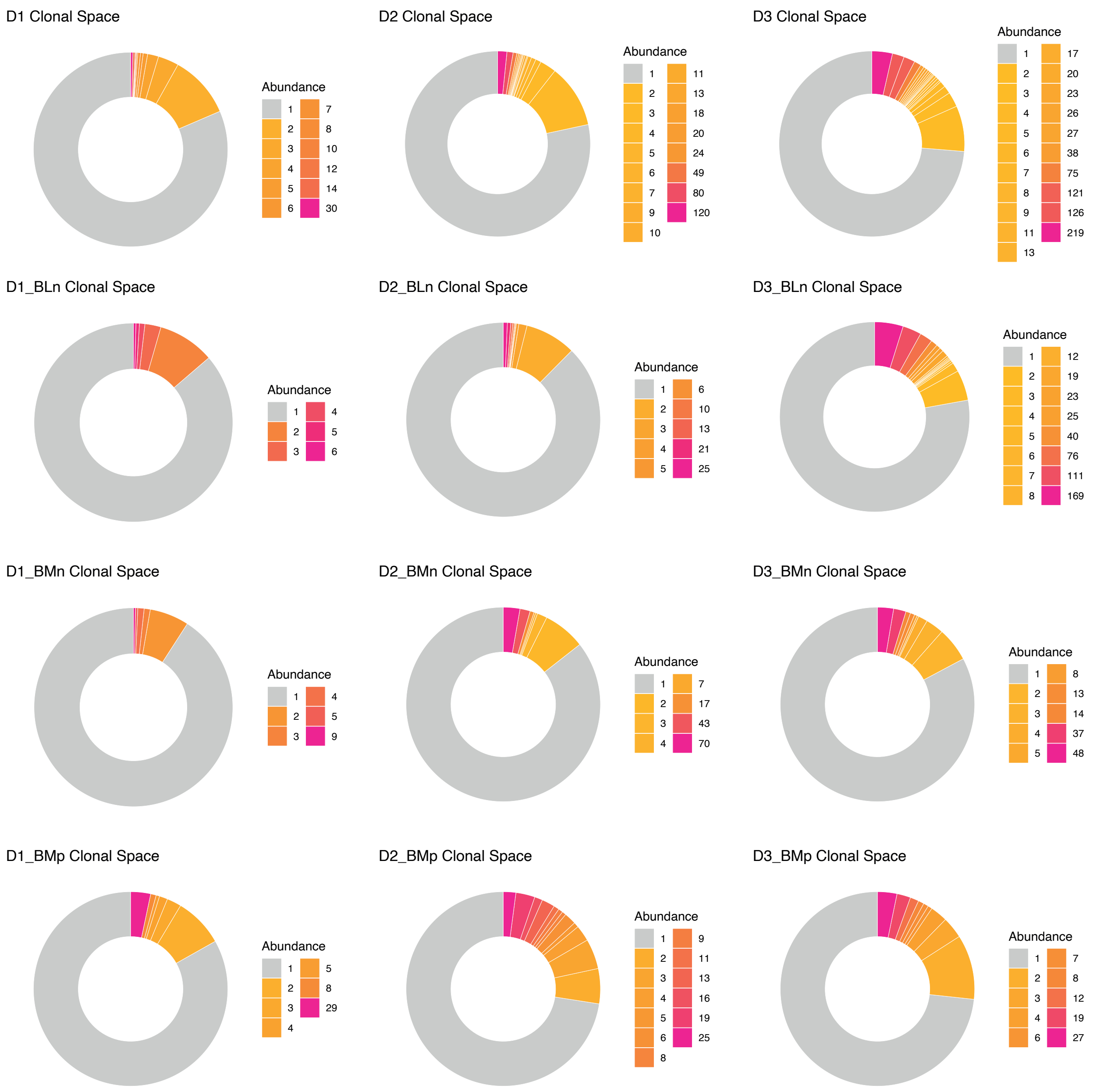

b

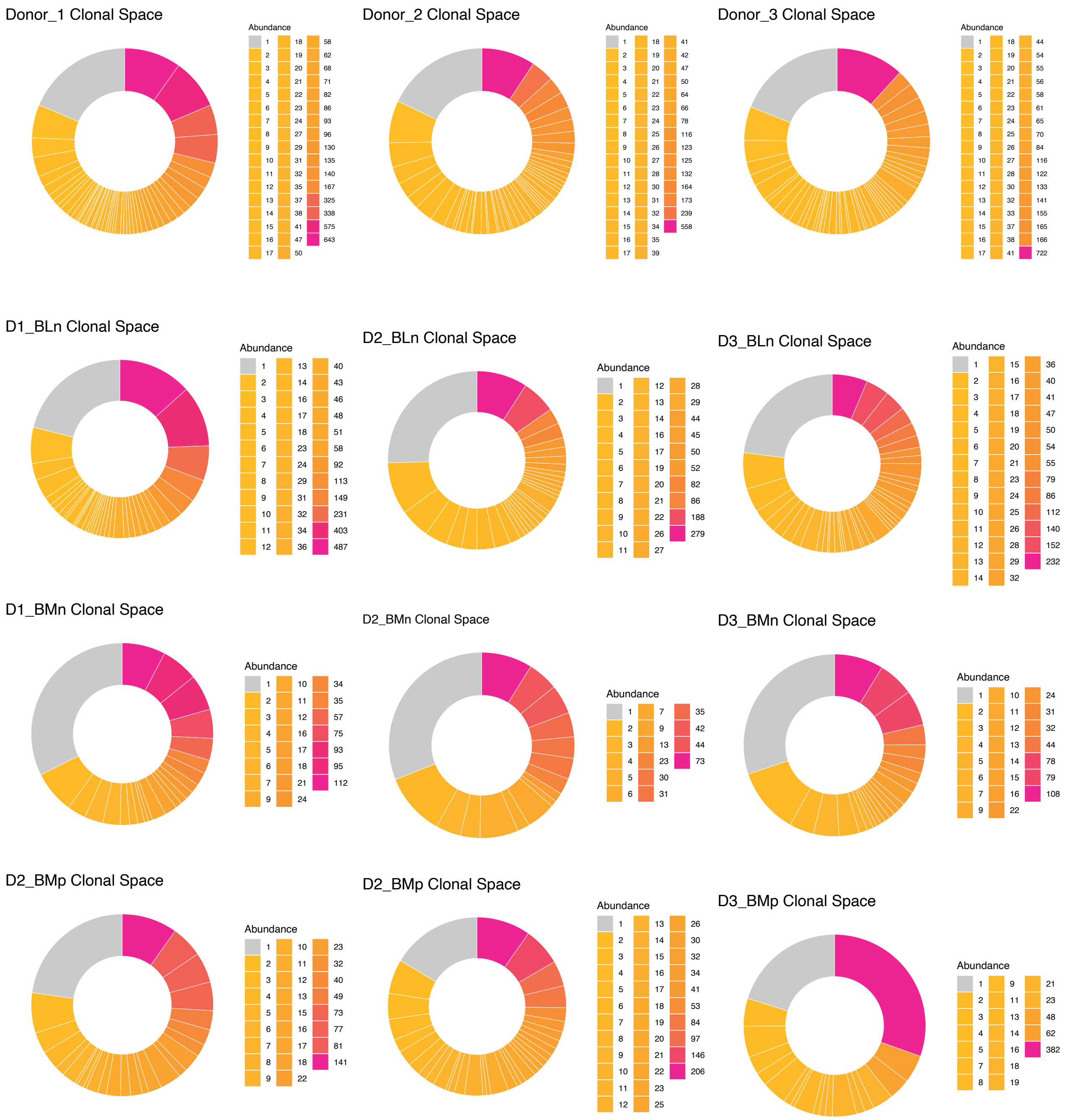

Supplementary Figure 4. Clonal space of CD4+ and CD8+ memory T cells. Abundance of each TCR is shown in the donut plots by color and proportional width according to the size of the clone in relation to the total TCRs. (a) CD4+ and (b) CD8+ TCR clonal spaces of the each donor (upper panels), followed by clonal spaces according to tissue origin. 2nd raw corresponds to blood, third raw to CD69- cells from the bone marrow and fourth raw to CD69+ cells from the bone marrow. D1-D3 corresponds to the donor ID, BLn corresponds to TBL, BMn to CD69- TBM and BMp to CD69+ TBM.

a

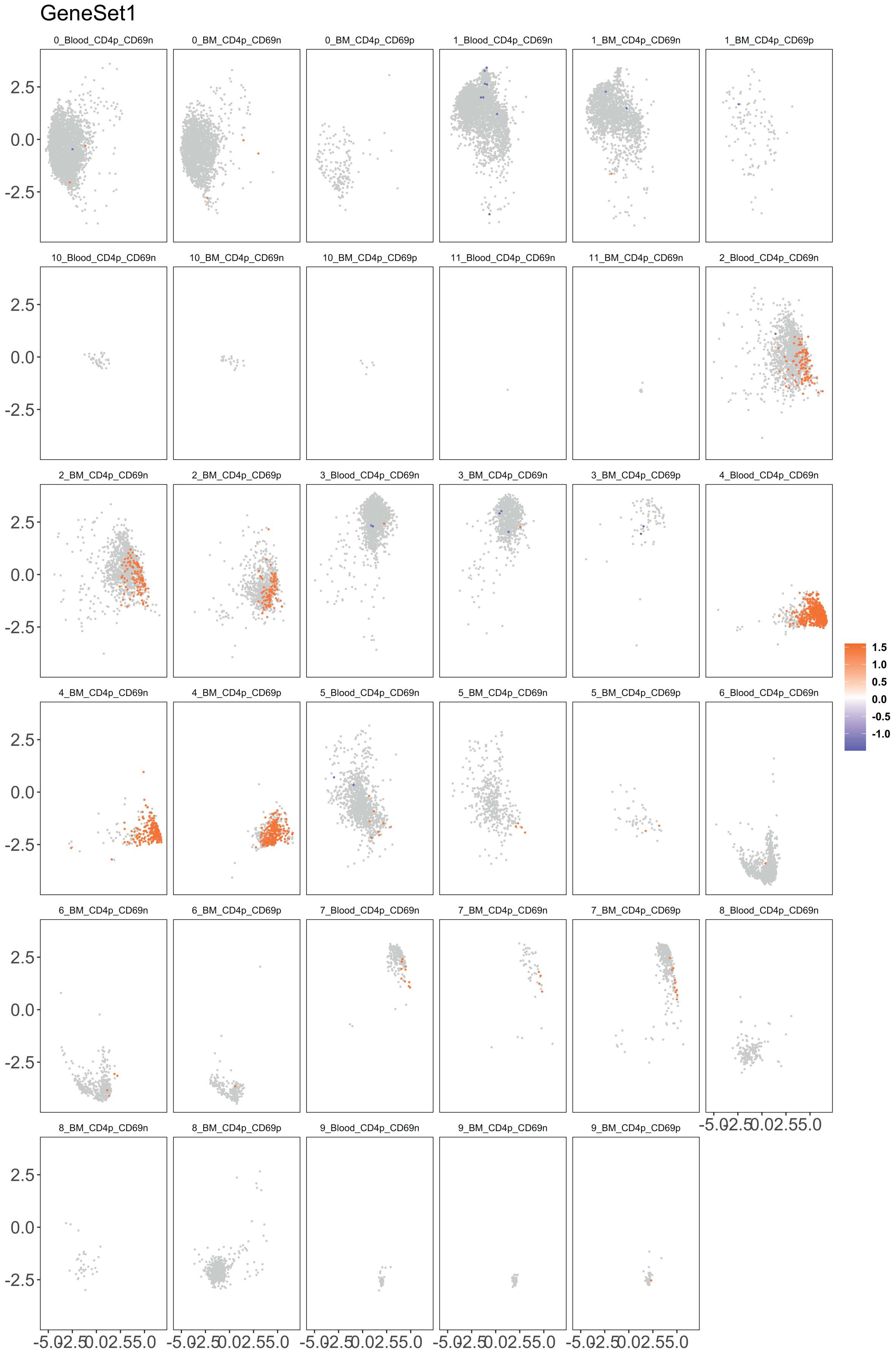
GeneSet1
0_Blood_CD4p_CD69n
0_BM_CD4p_CD69n
0_BM_CD4p_CD69p
1_Blood_CD4p_CD69n
1_BM_CD4p_CD69n
1_BM_CD4p_CD69p
10_Blood_CD4p_CD69n
10_BM_CD4p_CD69n
10_BM_CD4p_CD69p
11_Blood_CD4p_CD69n
11_BM_CD4p_CD69n
2_Blood_CD4p_CD69n
2_BM_CD4p_CD69n
2_BM_CD4p_CD69p
3_Blood_CD4p_CD69n
3_BM_CD4p_CD69n
3_BM_CD4p_CD69p
4_Blood_CD4p_CD69n
4_BM_CD4p_CD69n
4_BM_CD4p_CD69p
5_Blood_CD4p_CD69n
5_BM_CD4p_CD69n
5_BM_CD4p_CD69p
6_Blood_CD4p_CD69n
6_BM_CD4p_CD69n
6_BM_CD4p_CD69p
7_Blood_CD4p_CD69n
7_BM_CD4p_CD69n
7_BM_CD4p_CD69p
8_Blood_CD4p_CD69n
8_BM_CD4p_CD69n
8_BM_CD4p_CD69p
9_Blood_CD4p_CD69n
9_BM_CD4p_CD69n
9_BM_CD4p_CD69p
2.5
0.0
-2.5
-5.0 -2.5 0.0 2.5 5.0
1.5
1.0
0.5
0.0
-0.5
-1.0

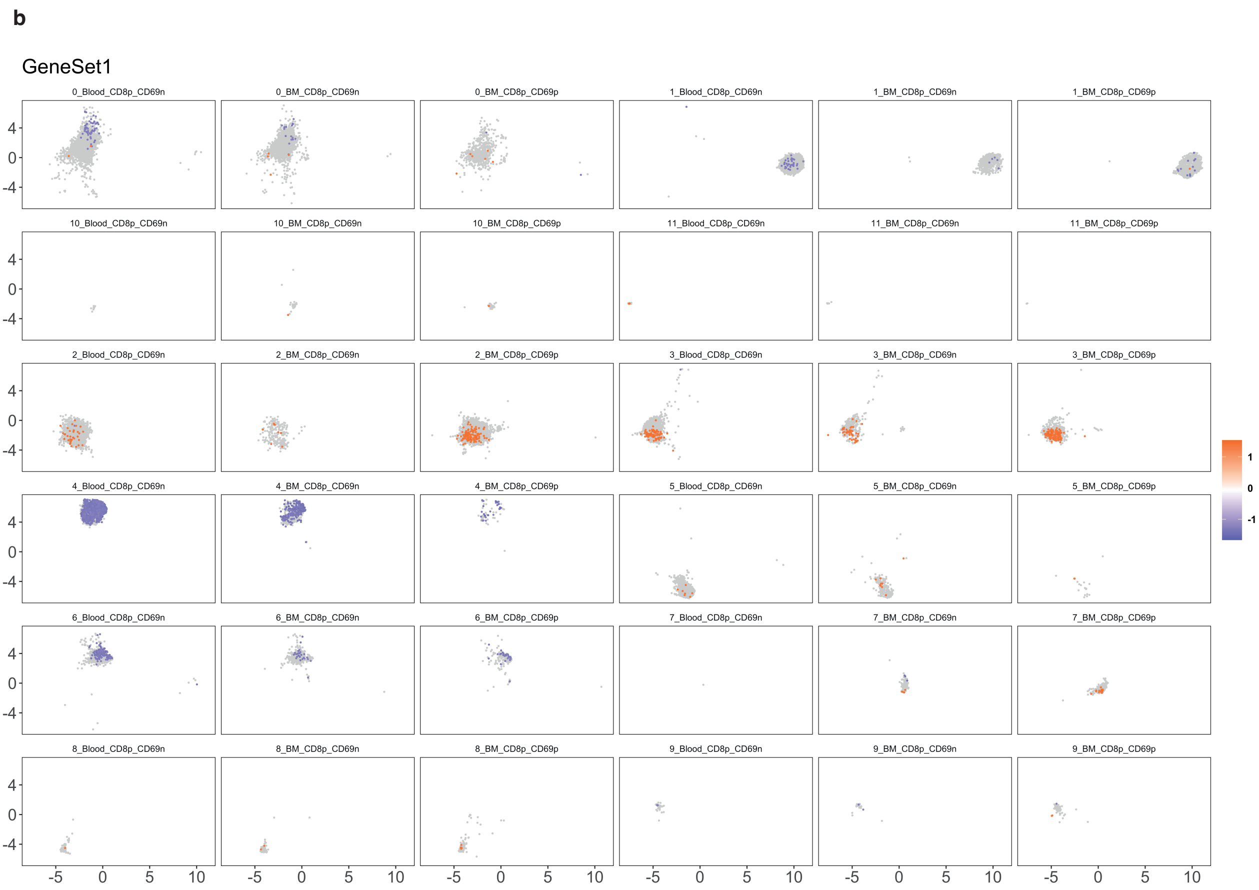

Supplementary Figure 5. Gene set enrichment analysis of Tr1-like cells. (a-b) UMAPs separated by cluster (first number in the panel title), tissue origin (blood and bone marrow (BM)) and CD69 cell surface expression(CD69+ as CD69p and CD69- as CD69n). CD4p corresponds to CD4+ cells, CD8p to CD8+ cells. Methods for the gene sets are reported elsewhere(36) and the genes for the gene set were defined according the gene list of Tr1-like cells in other publication(25).

a

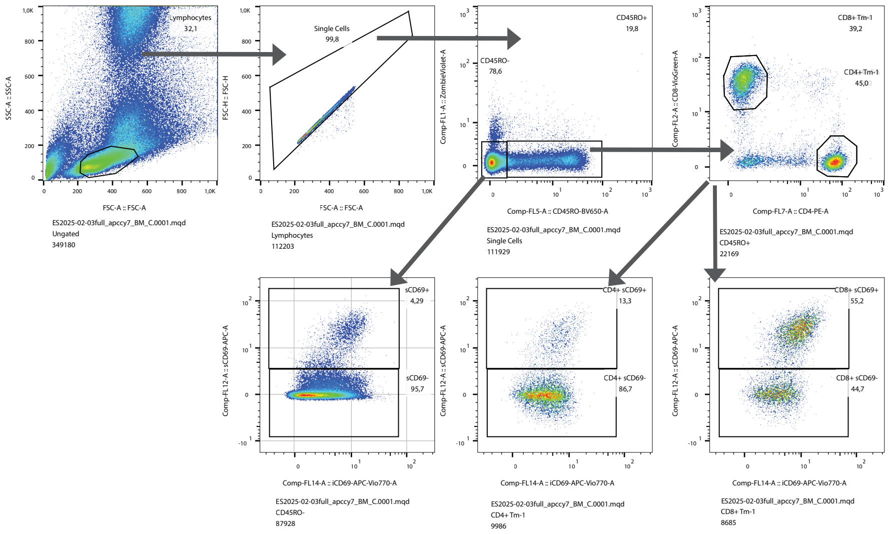

b

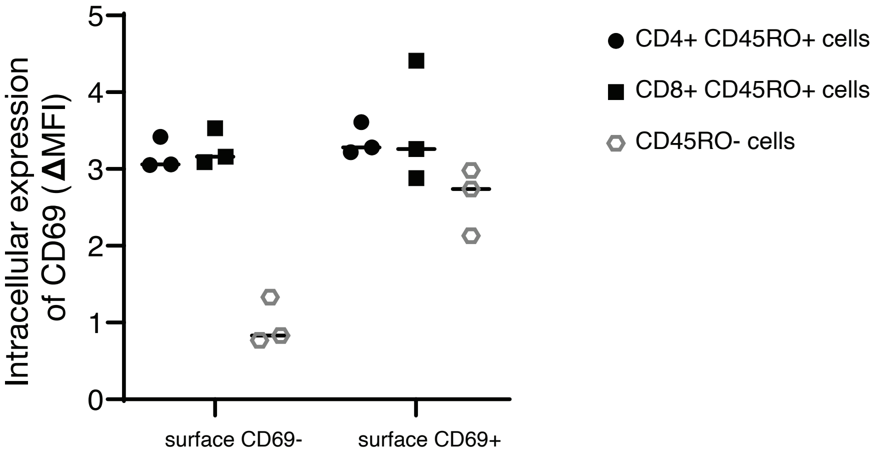

Supplementary Figure 6. Intracellular expression of CD69 protein in surface CD69+ and surface CD69- TBM. Cells were stained for surface CD69, then permeabilized or not with saponin, and stained for intracellular CD69. (a) the gating strategy for CD4+ and CD8+ CD45RO+ memory T cells and CD45RO- cells, expressing surface CD69 or not. (b) intracellular staining for CD69 of permeabilized versus non-permeablized cells as difference in mean fluorescence intensities (**Δ**MFI), and compared for surface CD69+ and surface CD69- cells.

SUPLEMENTARY INFORMATION

SUPLEMENTARY FIGURES

Supplementary Figure 1. Isolation and purification of memory T cells.
Supplementary Figure 2. Histograms comparing the number of observed overlapping clonotypes to the overlap of (1000x) random reshuffling of TCR clonotypes of all donors.
Supplementary Figure 3. CITE-seq antibody differential expression
Supplementary Figure 4. Clonal space of CD4+ and CD8+ memory T cells.
Supplementary Figure 5. Gene set enrichment analysis of Tr1-like cells.
Supplementary Figure 6. Intracellular expression of CD69 protein in surface CD69+ and surface CD69- T_{BM}.

SUPLEMENTARY TABLES

Supplementary Table 1. List of patients data
Supplementary Table 2. Antibody List
Supplementary Table 3. Tables of the difference between observed overlap and random overlap for each tissue per cluster.

SUPLEMENTARY DATA

Supplementary Data 1. Differential gene expression for each cluster. (a) CD4+ memory T cell data, in first tab, data obtained by considering min.pct as 0.5 and log2FC as 0.25; second tab min.pct= 0.1 and log2FC=0.3. (b) CD8+ memory T cell data, in first tab, data obtained by considering min.pct as 0.5 and log2FC as 0.25; second tab min.pct= 0.1 and log2FC=0.3.

Supplementary Data 2. Overlaps of TCR repertoires from different tissues within clusters by donors. Histograms for each cluster and donor, comparing the number of observed overlapping clonotypes to the overlap of (1000x) random reshuffling of TCR clonotypes for T_{BL} and CD69- T_{BM}, T_{BL} and CD69+ T_{BM}, CD69+ T_{BM} and CD69- T_{BM}, and the overlap of these three compartments together. The observed overlap is indicated by a black line and red number. The bar histogram indicates the random overlaps. The p value indicates the probability that observed and random overlap are the same. (a,b) CD4+ and CD8+ memory T cell data, where each tab corresponds to each donor. Data from the histograms is summarized in the shown matrix.

Supplementary Data 3. Differential gene expression within clusters by tissue origin. Tables with differentially expressed genes supporting volcano plots from Fig. 3. Obtained as described in the legend of Figure 3. Each tab contains the data of the volcanos from a to j.
